# Non-Immersive Virtual Reality-Based Therapy Applied in Cardiac Rehabilitation: A Systematic Review with Meta-Analysis

**DOI:** 10.3390/s24030903

**Published:** 2024-01-30

**Authors:** Ana Belén Peinado-Rubia, Alberto Verdejo-Herrero, Esteban Obrero-Gaitán, María Catalina Osuna-Pérez, Irene Cortés-Pérez, Héctor García-López

**Affiliations:** 1 Asociación de Fibromialgia de Jaén (AFIXA), C/Baltasar de Alcázar 5, 23008 Jaén, Spain; abpr0003@red.ujaen.es; 2 Department of Health Sciences, University of Jaén, Campus Las Lagunillas s/n, 23071 Jaén, Spain; eobrero@ujaen.es (E.O.-G.); mcosuna@ujaen.es (M.C.O.-P.); 3 Department of Nursing, Physiotherapy and Medicine, University of Almería, Ctra. Sacramento s/n, 04120 Almería, Spain; avh275@inlumine.ual.es (A.V.-H.); hector.garcia@ual.es (H.G.-L.)

**Keywords:** non-immersive virtual reality, active videogames, cardiac rehabilitation, aerobic capacity, anxiety, depression, quality of life, meta-analysis

## Abstract

Background: The aim of this systematic review with meta-analysis was to assess the effectiveness of non-immersive virtual reality (niVR) active videogames in patients who underwent cardiac rehabilitation (CR). Methods: A systematic review with meta-analysis, according to the PRISMA guidelines and previously registered in PROSPERO (CRD42023485240), was performed through a literature search in PubMed (Medline), SCOPUS, WOS, and PEDro since inception to 21 November 2023. We included randomized controlled trials (RCTs) that assessed the effectiveness of an niVR intervention, in comparison with conventional CR and usual care, on aerobic capacity and cardiovascular endurance (physical function), anxiety, depression, and quality of life (QoL). The risk of bias in individual studies was assessed using the Cochrane risk of bias tool. Effect size was estimated using Cohen’s standardized mean difference (SMD) and its 95% confidence interval (95% CI) in a random-effects model. Results: Nine RCT that met the inclusion criteria were included in the meta-analysis. The meta-analysis showed a moderate-to-large effect favoring niVR active videogames included in CR in increasing aerobic capacity and cardiovascular endurance (SMD = 0.74; 95% CI 0.11 to 1.37; *p* = 0.021) and reducing anxiety (SMD = −0.66; 95% CI −1.13 to −0.2; *p* = 0.006). Only 4.8% of patients reported adverse events while performing niVR active videogames. Conclusions: Inclusion of niVR active videogames in CR programs is more effective than conventional CR in improving aerobic capacity and cardiovascular endurance and in reducing anxiety.

## 1. Introduction

Cardiovascular diseases (CVD) comprise a group of disorders that affect the heart and blood vessels and include high blood pressure, cerebrovascular disease, heart failure, coronary heart disease, peripheral vascular disease, rheumatic heart disease, cardiomyopathies, and congenital heart diseases, among others [1,2]. In 2019, 17.9 million deaths were attributed to CVD (32% of all deaths worldwide, especially in developing countries), making them the main cause of death worldwide [2]. Their prevalence is expected to increase due to the sedentary habits of the population [3]. CVD manifests with numerous signs and symptoms, the most common being chest discomfort or pain, difficulty breathing, fatigue when walking or performing any activity, high blood pressure and edema in the lower extremities [4,5]. These symptoms diminish the physical and psychosocial health of patients, leading to loss of functional capacity, anxiety, or depression, which translates into a loss of quality of life (QoL) for these patients and their caregivers [6], in addition to representing a major economic burden on healthcare and social systems [7].

Control of CVD risk factors through drugs [8,9,10], physical exercise [11], and maintaining a healthy diet and an active lifestyle are the most commonly used therapeutic measures in these patients [12]. Once CVD has been diagnosed, or after cardiovascular surgery, cardiac rehabilitation programs (CRP) are essential and commonly used [13,14]. CRP is defined as an integral and multidisciplinary intervention composed of preventive strategies based on physical exercise [15], the reduction of CVD risk factors, and long-term care for the cardiac patients throughout hospitalization and discharge phases (outpatient and maintenance) [15,16,17]. It is the main intervention used as secondary prevention [18]. CRP programs incorporate essential elements, such as nutrition, psychological well-being, and education, that provide health benefits and economic advantages by reducing hospitalization times and medical expenses, improving patient prognosis, and reducing mortality by up to 53% [19,20]. However, despite the benefits of CRP, patient participation and adherence rates in these programs remains low, around 30% [21], with a dropout rate between 12% and 56% [22]. The reasons for this low rate may include lack of awareness on the part of patients, lack of economic resources and social support [19,22], as well as the perception of therapy as monotonous and based on poorly challenging and motivating exercises. In this context, the inclusion of new technologies that gamify CRP protocols, making them more engaging, could improve adherence and motivation [23].

New technologies in the field of health have experienced a great boom in recent decades, especially virtual reality (VR) devices. In the management of patients with CVD, CRP have also included exercises with VR devices and videogames (exergames and serious games). VR devices allow the creation of virtual environments that simulate real life, and in which the user feels immersed (presence) and can interact with virtual elements (immersion) [24,25]. Depending on the level of presence and immersion, VR devices can be immersive, semi-immersive or non-immersive (niVR) [26], with the latter being the most commonly assessed in literature. The niVR devices use computer software or video consoles to project virtual environments on bi-dimensional screens, allowing interaction between persons and virtual objects using a joystick or keyboard [27]. These devices offer advantages such as ease of accessibility and low-cost, although the latter depends on the type of niVR device used, especially their trademarks [28]. Commercial video consoles such as Nintendo Wii or Switch and Microsoft Xbox, are the most widely used niVR devices in rehabilitation [29,30], combining gamification with active and enjoyable physical exercises that promote patients’ motivation [31]. These most common niVR devices allow the interaction between the patient and the virtual environment using a virtual avatar that reproduces the body movements using different motion sensing input devices, such as the Wii Remote or a Kinect^®^ sensor. To interact with Nintendo videogames, the Wii Remote, also called Wiimote (Nintendo, Redmond, WA, USA) was developed and has been used since 2006 [32]. The Wii Remote is a wireless (via Bluetooth connectivity) handheld device that consists of a speaker, a vibrator, and a high-speed infrared camera. All of these allow one to capture and generate an image of a patient’s real movements in the virtual environment [33]. Alternatively, the Microsoft Kinect^®^ sensor, developed and marketed by Microsoft Corporation in 2010 (Microsoft Corporation, Redmond, WA, USA) [34], is a low-cost motion capture and analysis system that has been integrated in videogames and serious games for clinical and rehabilitation purposes [35]. Kinect^®^, widely used in motor and gait rehabilitation, allows the real-time and non-invasive capture of patients’ real movements, generating a three-dimensional skeletal model that reproduces real movements in the virtual world [36]. These niVR devices, with their sensors, allow the patient to move and interact with objects in the virtual world, enabling them to perform physical training or real tasks that are simulated in the virtual environment. NiVR devices encourage physical exercise that is more engaging and challenging [37], becoming more motivating and less monotonous, and ensuring that the patient perceives less sensation of effort or fatigue [38], which in turn increases adherence and patient satisfaction, potentially leading to satisfactory functional recovery [39].

In recent years, the use of niVR active videogames in CRP has increased. To date, four reviews (one qualitative review [16] and three meta-analyses [40,41,42]) have assessed the effect of all VR devices as therapeutic tools to be included in CRP. However, the effectiveness (or non-effectiveness) of VR devices on specific variables such as functional capacity or cardiovascular endurance, anxiety, depression, or QoL is not clear. It is important to note some limitations of the previous reviews that could reduce the statistical power, quality of evidence, and generalization of their findings. These include (1) the low number of studies included per meta-analysis (four as a maximum), (2) the inclusion of studies with repeated samples for the same analysis (i.e., combining data of the different dimensions of QoL to obtain a common result), and (3) measuring different constructs (anxiety and depression) in the same meta-analysis. Additionally, the inclusion of studies on both immersive and niVR interventions in these reviews could lead to heterogeneity between studies, potentially over/underestimate the original findings. Therefore, aiming for a more homogenous analysis and recognizing that niVR devices are the most used, we compiled all randomized controlled trials (RCT) published to date to assess the effectiveness of niVR active videogames in improving functional capacity, anxiety, depression, and different dimensions of QoL in patients undergoing CRP. As a secondary objective, we aim to determine the proportion of adverse events experienced by patients during exposure to niVR active videogames.

## 2. Materials and Methods

### 2.1. Study Design and Protocol

This systematic review with meta-analysis was performed using the recommendations of the Preferred Reporting Items for Systematic Reviews and Meta-Analyses (PRISMA) statement [43], and the Cochrane Handbook for Systematic Reviews of Interventions [44]. Additionally, the protocol of this review has been previously registered in the PROSPERO database (CRD42023485240).

### 2.2. Literature Search and Databases

A literature search was carried out by two authors (A.B.P.-R. and H.G.-L.), independently, since inception to November 21 2023, in various health science databases, including PubMed (Medline), SCOPUS, Web of Science (WOS), CINAHL Complete, SciELO, and the Physiotherapy Evidence Database (PEDro). In addition, the reference lists of previous published studies related to our topic, document experts, or congress abstracts were reviewed in detail. To build the research question, the PICOS method proposed by the Cochrane Collaboration was used, as follows [45]: population (patients with CVD); intervention (niVR active videogames), comparison (other interventions), outcomes (aerobic capacity, anxiety, depression, and QoL), and study design (randomized controlled trials (RCT) or pilot RCT). However, to increase the sensitivity of our literature search, and to avoid missing potential studies, we only combined two conditions (population and intervention). Based on the medical subject headings (MeSH), the terms used in the search strategy were “cardiac rehabilitation”, “virtual reality”, “virtual reality exposure therapy”, and “exergaming”, along with their synonyms. The boolean operators “AND/OR” were used to combine the terms in the search. Finally, no language or publication date filters were used. This phase was supervised by a third author with expertise in search strategy (I.C.-P.). Table 1 shows the search strategy used in each of the databases explored.

### 2.3. Study Selection: Inclusion and Exclusion Criteria

Two authors (A.B.P.-R. and H.G.-L.), independently and carefully reviewed all records retrieved in the literature search by title and abstract. When a record was identified as potentially includable in the review, it was examined by two authors. Disagreements and uncertainties were resolved by a third author (I.C.-P.).

To include a study in the present review, it had to meet all of the following inclusion criteria, which were related to the PICOS criteria: (1) use RCT or RCT pilot, (2) be composed of patients with CVD divided into at least two groups, with (3) one group receiving niVR active videogames as an experimental intervention and (4) in comparison with another group that received a therapy different from niVR, and should (5) provide quantitative data of the variables of interest (aerobic capacity and cardiovascular endurance, anxiety, depression and QoL) eligible for inclusion in the quantitative synthesis (meta-analysis). The exclusion criteria proposed were as follows: (1) studies providing quantitative data that could not be used in a meta-analysis and (2) studies with a sample composed of patients with different diseases, not exclusively a sample of patients with CVD.

### 2.4. Data Extraction

Two authors (A.B.P.-R. and A.V.-H.), independently extracted the data of the studies included in the review, using a standardized Microsoft Excel data-collection form designed for this research. From each study, the following data were extracted: (1) general characteristics of the study (authors, years of publication, country, blinding, setting, funding, total sample size, CVD diagnosed, and number of groups per study), (2) characteristics of each group (number of participants in each group, age and gender), (3) characteristics of each intervention (type of intervention and protocol administration and, for the niVR intervention, we collected the niVR device used), and (4) data related to variables (name of the variable, test used to assess it, and quantitative data of post-intervention assessment (mean and standard deviation)) and to the adverse events (number of participants suffering adverse events in all patients exposed to niVR).

### 2.5. Variables

The variables assessed in this systematic review with meta-analysis were aerobic capacity, anxiety, depression and QoL. Specifically, for QoL, five dimensions were assessed: physical, mental, social, vitality, and overall perceived health.

### 2.6. Risk of Bias and Quality of Evidence Assessments

The risk of bias of the individual studies included in the review was evaluated with the Cochrane Collaboration risk of bias (CrOB) tool, using the software Review Manager (RevMan) version 5.4 (The Nordic Cochrane Centre, The Cochrane Collaboration, Copenhague, Denmark) [44,46]. This scale, comprising seven items, is able to identify the following biases in RCT: selection, performance, detection, attrition, reporting, and others. This tool labels risk as low, uncertain (when studies do not provide information about this), or high risk of bias. Each domain is rated according to compliance with the evaluation criteria, assigning a color, green for low risk of bias (when the criteria are appropriately met), red for high risk of bias (when the criteria are not met), and yellow for unclear risk of bias (when there is a lack of information to determine the compliance with these criteria). For each study, a final score calculation was performed, which was converted into a percentage to obtain a summary measure of overall risk of bias. Trials with an overall score of 75% or more were classified as having a low risk of bias, those with an overall score between 50% and 74% were classified as having an unclear medium risk of bias, and trials with an overall score of 75% or more were classified as having a low risk of bias.

To assess the quality of evidence of the meta-analyses’ results, the Grading of Recommendations Assessment, Development, and Evaluation (GRADE) and the Meader’s checklist were used [47,48]. Five items are considered when reporting the quality of evidence: risk of bias in individual studies, inconsistency, indirectness, imprecision, and the risk of publication bias. For each item or factor that is not met, the quality of evidence will be downgraded one level. Finally, the quality of evidence could be (1) high, indicating robust findings; (2) medium, when our results might change with new research; (3) low, indicating a low level of confidence in the effect; and (4) very low, when any estimate of the effect is highly uncertain.

Two authors (E.O.-G. and M.C.O.-P.) were independently responsible for assessing the risk of bias in individual studies and the quality of evidence of the main findings. Discrepancies were resolved through consultation with a third author (H.G.-L.).

### 2.7. Statistical Analyses

The statistical analysis (meta-analysis) was performed using Comprehensive Meta-Analysis version 4.0 (Biostat, Englewood, NJ, USA) [49,50] by one author (E.O.-G.). It is important to note that a meta-analysis was only conducted when at least three comparisons (k = 3) per variable were available, with the goal of obtaining findings with a minimum of statistical power. In addition, to reduce the impact of the heterogeneity of intervention protocols between studies, all analyses were applied under the random-effects model of Dersimonian and Laird [51]. Pooled effect or effect size were estimated using the Cohen’s standardized mean difference (SMD) and its 95% confidence interval (95% CI) [52], and the results are displayed in the forest plots [53]. In these forest plots, red diamonds represent the overall results of the meta-analysis, either from the subgroup analysis performed (subtotals) or from the set of all groups (total). The center of the diamond is the overall effect value and the width represents the overall confidence interval. The difference between the intervention and control groups can be considered statistically significant if the diamond is clearly positioned to one side of the reference line, but if it crosses it or just touches it, no conclusions can be drawn from that point in one direction. On the other hand, the black square represents the individual effect of each study in the meta-analysis and its horizontal black line represents the confidence level of this effect. Effect size can be interpreted as null (SMD = 0), low (SMD = 0.1–0.39), medium (SMD = 0.4–0.79), and large (SMD > 0.8) [54]. To assess the proportion of patients reporting adverse events, a meta-analysis of proportions estimating the event rate was conducted. The risk of publication bias was assessed with the *p*-value for Egger’s test and the visualization of the funnel plots (*p* < 0.1 and a clearly asymmetric funnel plot indicates a risk of publication bias) [55,56]. In addition, we used the trim-and-fill method to estimate the adjusted effect of the intervention considering the possible risk of publication bias, to determine whether the original effect was under- or overestimated [57,58]. Finally, heterogeneity was calculated using degree of inconsistency (I^2^), the Q-test, and its *p*-value (*p* < 0.05 is associated with a risk of heterogeneity or inconsistency). Heterogeneity can be classified as null (I^2^ = 0%), low (I^2^ = 25%), moderate (I^2^ = 50%), or large (I^2^ > 75%) [59,60].

As an additional analysis, we conducted a single-group proportional meta-analysis (using the total sample size and the number of cases of adverse events) to estimate the proportion of adverse events during niVR exposition. Sensitivity analysis using the leave-one-out method (or also called “one study removed method”) was used to assess the contribution of each study to the global effect.

## 3. Results

### 3.1. Study Selection

**Figure 1:**
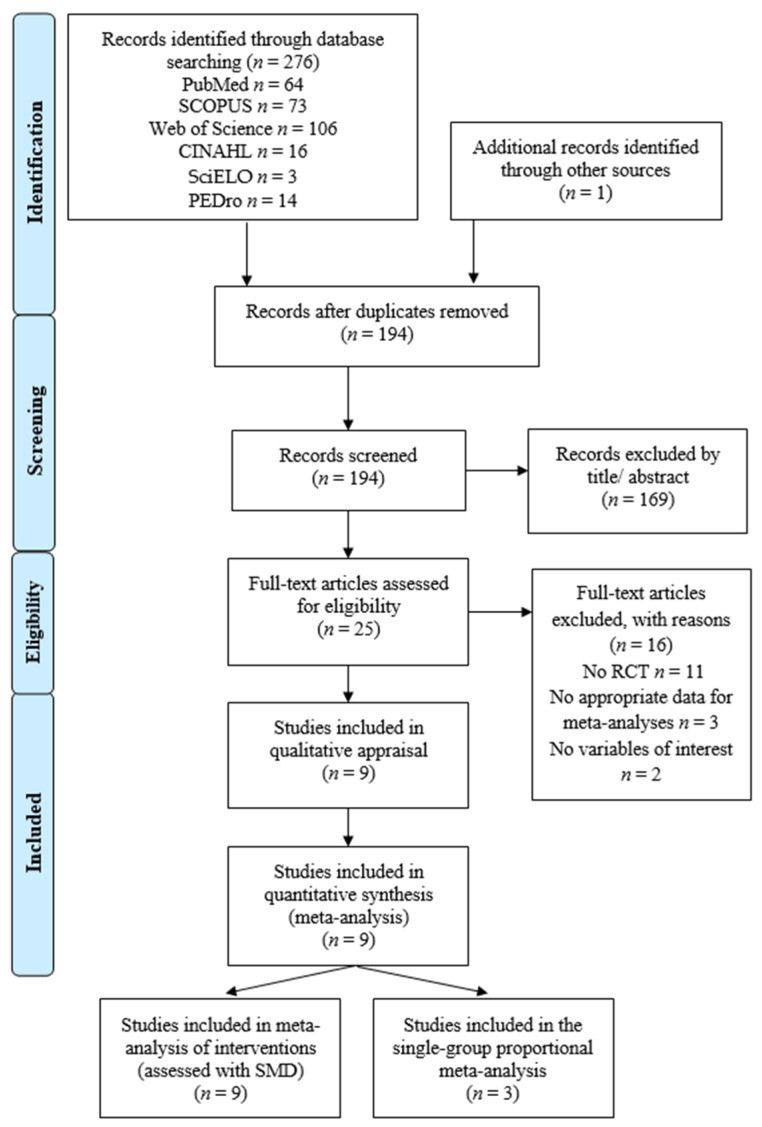
PRISMA flow diagram.

Two hundred and seventy-seven records were retrieved by initial searches (276 from databases and 1 from a reference list of previous papers published). When duplicates were removed, 194 references were reviewed by title/abstract. Of these, 169 references were excluded by title/abstract and 16 for not meeting the inclusion criteria (reasons in Figure 1). In the end, nine RCTs [61,62,63,64,65,66,67,68,69] were included in the present systematic review with meta-analysis of interventions, and three of these nine studies [64,66,67] were included in the proportional meta-analysis to estimate the proportion of adverse events during niVR exposition. The PRISMA flow diagram, in Figure 1, summarizes this process.

### 3.2. Characteristics of the Studies Included in the Review

The RCTs included, carried out between 2013 and 2023, provided data from 848 patients with CVD from Brazil [61,62,67], Spain [63], United States [64], Portugal [68], Ireland [66], Thailand [69], and one multicentre RCT from Sweden, Italy, Israel, Netherlands, Germany, and the United States [65]. The mean age of the included patients was 60.2 ± 5.8 years old (67% males). The studies included reported data from patients diagnosed with arterial hypertension [61], heart failure [63,65], valve failure, or coronary artery bypass [62,66,69] and mixed CVD [64,67,68] in phase I [62,66,69], II [61,63,64], and III [65,68]. Four hundred fourteen patients received niVR active videogames (mean age of 58.7 ± 6.4 years old), and 434 patients comprised the comparison group, receiving other interventions such as physical therapy, exercise, or usual care. All RCTs included provided data from post-immediate assessment (after finishing the intervention). Finally, six studies received external funding to conduct the study [61,62,64,65,67,69]. Table 2 shows the main characteristics of the studies included in the meta-analysis.

### 3.3. Assessment of Risk of Bias in the Studies Included in the Review

**Figure 2:**
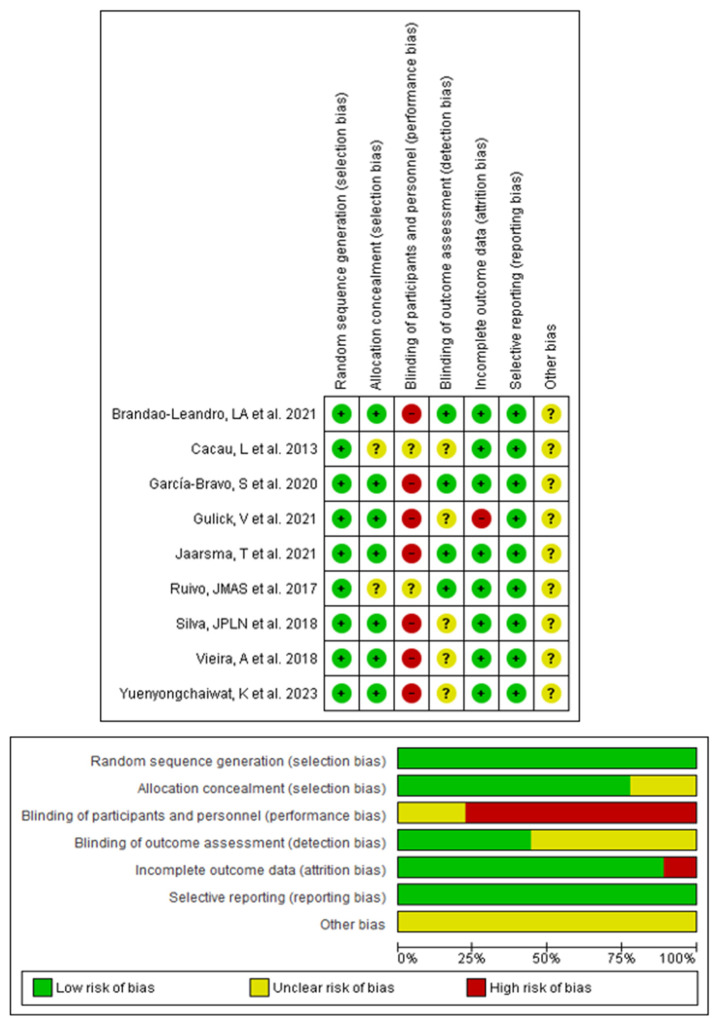
Cochrane risk of bias tool for the risk of bias in individual studies [61,62,63,64,65,66,67,68,69].

Figure 2 shows the risk of bias in individual studies assessed with the CRoB. Four RCT included in this meta-analysis showed low risk of bias [61,63,65,66], and in five RCTs, the risk of bias was unclear [62,64,67,68,69]. The mean risk of bias score was 72% ± 6.8% (Supplementary Table S1 that provides the mean risk of bias of each study included [61,62,63,64,65,66,67,68,69]). All studies demonstrated a low risk of bias in the domains of randomization sequence and selective reporting of results, and seven studies showed adequate allocation concealment [61,63,64,65,67,68,69]. In seven studies, participants and therapists were not blinded [61,63,64,65,67,68,69], and in two studies, the risk was unclear [62,66], indicating the importance of considering performance bias for all studies. Regarding whether evaluators were blinded, in four RCTs the risk was low [61,63,65,66] and unclear in the others [62,64,67,68,69]. Only one RCT [64] reported attrition bias.

### 3.4. Outcome Synthesis and Measurements

#### 3.4.1. Aerobic Capacity and Cardiovascular Endurance (Physical Function)

**Figure 3:**
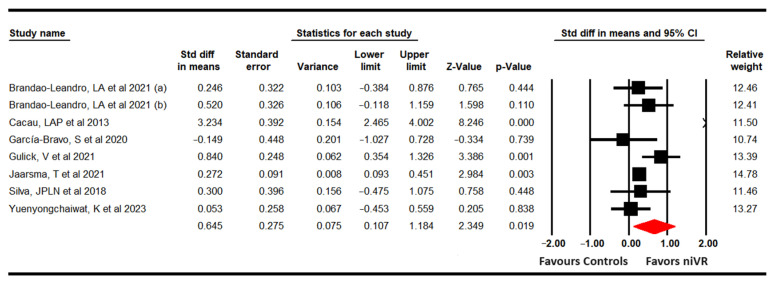
Forest plot for aerobic capacity [61,62,63,64,65,67,69].

Seven studies with eight independent comparisons [61,62,63,64,65,67,69] provided data from 812 participants (101.5 per comparison) to assess the effectiveness of niVR active videogames on aerobic capacity and cardiovascular endurance using the 6-Minutes Walking Test (6-MWT). The meta-analysis showed, with low quality of evidence, a large effect (SMD = 0.65; 95% CI 0.11 to 1.18; *p* = 0.019) favoring niVR, compared with classical CRP and usual care (Figure 3). In addition, our analysis reported that carrying out CRP with niVR active videogames could increase the final distance traveled in the 6-MWT by 49.32 m (95% CI 41.9 to 56.7; *p* < 0.001). However, the risk of publication bias (Egger *p* = 0.07) suggested it might be responsible for underestimating the original pooled effect, as trim-and-fill estimation reported an adjusted effect of 1 (95% CI 0.4 to 1.6), taking into account this potential risk of publication bias (Supplementary Figure S1). Finally, heterogeneity was moderate (I^2^ = 39.3%; Q = 12.5; df = 7; *p* = 0.08) and sensitivity analysis did not reveal any variation using the leave-one-out method.

#### 3.4.2. Anxiety

**Figure 4:**
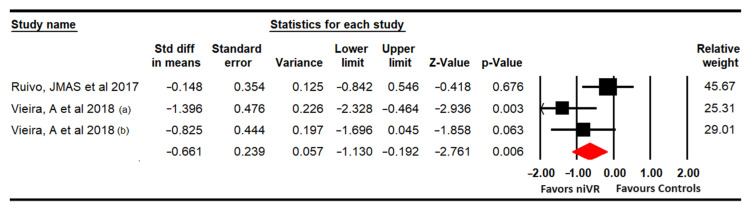
Forest plot for anxiety [66,68].

The effect of niVR active videogames on improving anxiety was assessed with data from 2 studies with 3 independent comparisons that reported data from 76 participants (25.3 per comparison) using the anxiety dimensions of the Hospital Anxiety and Depression Scale (HADS) [66] and the Depression, Anxiety, Stress Scales (DASS-21) [68]. Our findings show, with very low quality of evidence, a medium effect (SMD = −0.66; 95% CI −1.13 to −0.2; *p* = 0.006) (Figure 4) without risk of publication bias (Egger *p* = 0.24) or heterogeneity (I^2^ = 0%; Q = 1.93; df = 2; *p* = 0.38). Sensitivity analysis did not reveal any significant variations.

#### 3.4.3. Depression

**Figure 5:**
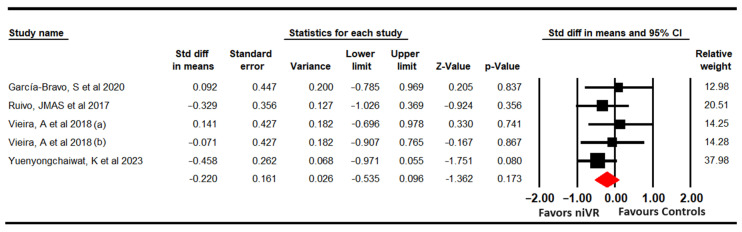
Forest plot for depression [63,66,68,69].

Four studies with 5 independent comparisons provided data from 156 participants (31.2 per comparison) using the Beck Depression Inventory (BDI) [63], the depression dimensions of the HADS [66], the DASS-21 [68], and the Patient Health Questionnaire 9 (PHQ-9) [69], respectively. No statistically significant differences (SMD = −0.22; 95% CI −0.54 to 0.1; *p* = 0.173) were found (Figure 5), with no evidence of heterogeneity (I^2^ = 0%; Q = 2.24; df = 4; *p* = 0.7).

#### 3.4.4. Quality of Life

**Figure 6:**
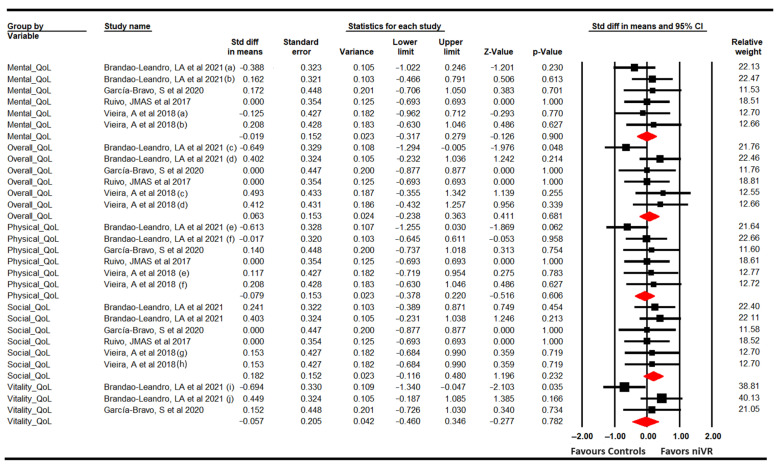
Forest plot for quality of life [61,63,66,68].

The studies included in this meta-analysis provided data by which to assess the following dimensions of quality of life: physical [61,63,66,68], mental [61,63,66,68], social [61,63,66,68], vitality [61,63], and overall perceived health [61,63,66,68]. Thus, a separate meta-analysis was conducted for each dimension. Our findings show that no statistically significant differences were found between niVR and controls for physical (k = 6; *n* = 174; SMD = −0.08; 95% CI −0.38 to 0.22; *p* = 0.61; I^2^ = 0%; Q = 3.64; df = 5; *p* = 0.6), mental (k = 6; *n* = 174; SMD = −0.02; 95% CI −0.32 to 0.28; *p* = 0.9; I^2^ = 0%; Q = 2.2; df = 5; *p* = 0.82) and social quality of life (k = 6; *n* = 174; SMD = 0.18; 95% CI −0.11 to 0.48; *p* = 0.232; I^2^ = 0%; Q = 0.94; df = 5; *p* = 0.98), vitality (k = 3; *n* = 98; SMD = −0.06; 95% CI −0.46 to 0.34; *p* = 0.782; I^2^ = 68.5%; Q = 6.4; df = 2; *p* = 0.04), and overall perceived health (k = 6; *n* = 174; SMD = −0.06; 95% CI −0.24 to 0.36; *p* = 0.68; I^2^ = 29.3%; Q = 7.4; df = 5; *p* = 0.19) (Figure 6).

#### 3.4.5. Proportion of Adverse Events during niVR Exposure

**Figure 7:**
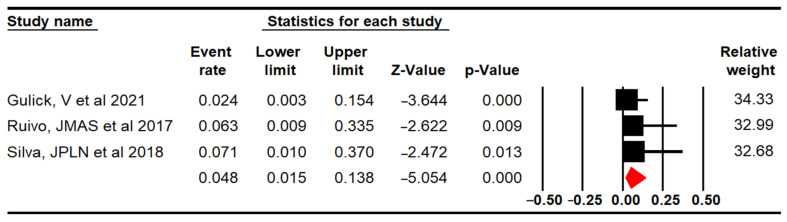
Forest plot for proportion of the adverse events reported [64,66,67].

The proportion of adverse or unwanted events in the niVR active videogames group was estimated with data from three RCTs with three independent comparisons [64,66,67]. The meta-analysis showed that only 4.8% (95% 1.5 to 13.8; *p* < 0.001) of patients who underwent niVR active videogames could potentially suffer adverse events (Figure 7), with cybersickness, dyspnea, and fatigue being the most reported. No risk of publication bias (Egger *p* = 0.34) and no evidence of heterogeneity (I^2^ = 0%; Q = 0.72; df = 2; *p* = 0.7) were observed, and sensitivity analysis showed a similar contribution of all studies to the global proportion.

## 4. Discussion

The present systematic review with meta-analysis was conceived with the aim of retrieving all previous RCTs regarding the effectiveness of niVR active videogames in CRP, especially to improve aerobic capacity, anxiety, depression, and different dimensions of the QoL. Additionally, as a secondary objective, we aimed to report the most common adverse events experienced by these patients and to estimate the proportion of these unwanted effects during CRP. To differentiate our meta-analysis from the previously published reviews [16,40,41,42], we only assessed the effectiveness of niVR active videogames or devices, as this modality of VR is the most widely published to date. This reduces the heterogeneity between therapies, as all therapies in the studies included use the same modality of VR, increasing the precision and generalization of our findings. Thus, our sensitive literature search strategy recovered nine unique RCTs published with data using niVR in CRP that provide data from 848 patients (60.2 ± 5.8 years) involved in CRP. Compared with previous reviews, the present meta-analysis is the first to exclusively assess the effect of niVR in CRP. Although previous meta-analyses, even including studies with interventions based on immersive and niVR, included a smaller number of studies than ours (*n* = 8 [40], *n* = 7 [41,42]) our results enhance the generalizability and quality of evidence of the studies previously published.

The first relevant finding in our meta-analysis is that niVR active videogames are effective (SMD = 0.65) in improving aerobic capacity and cardiovascular endurance (physical function) in these patients, with data from 812 participants from 7 studies [61,62,63,64,65,67,69]. However, the effect is 53% greater (adjusted SMD = 1), with no risk of publication bias assessed with trim-and-fill estimation, demonstrating that niVR is an effective therapy to be used in CRP to improve aerobic capacity. In previous reviews, it was not found to be clear whether niVR would be effective in improving this variable, as Blasco-Peris et al. (2022) [40] and Bashir et al. (2023) [42], with data from four and two studies, respectively, did not find a statistically significant effect on aerobic capacity, contrary to the meta-analysis of Chen et al. (2022) [41]. Our findings, agreeing with Chen et al. (2022), are more robust, as we included five more RCTs [61,63,64,67,69] in our meta-analysis and the level of statistical heterogeneity was lower, thereby enhancing the quality of the evidence. Moreover, we estimated that, compared with conventional approaches, persons who performed niVR active videogames increased the total distance in the 6-MWT by 49.32 m. This could be assessed as all RCTs included provided data using the same test (6-MWT) for this variable. This enabled us to compare our findings with the minimally clinically important difference (MCID) in 6-MWT, whose value is between 14 and 30.5 m in multiple patients groups (CVD and pulmonary diseases) [70], and about 36 m in patients with chronic heart failure [71]. This suggests that including niVR active videogames or devices in CRP is indeed effective in increasing the aerobic capacity and cardiovascular endurance of these patients, being able to sufficiently exceed the MCID for the 6-MWT.

Anxiety and depression are two common symptoms that negatively affect patients [72]. About 32.5% and 17.5% of patients with CVD reported anxiety and depression, respectively [73]. This review shows that niVR active videogames are effective in reducing anxiety (SMD = −0.66), but no statistically significant differences were found in reducing depression symptoms. These findings are not clearly comparable with any previous reviews. A study by Blasco-Peris et al. (2022), which includes in the same analysis (named “mental health”) data from anxiety and depression, did not find an effect [40]. Additionally, a meta-analysis by Chen et al. (2023), which included studies using immersive and niVR, found no effect on anxiety, but did find an effect on depression. However, in their last meta-analysis on anxiety, one study was excluded though it could have been included [66], while for depression, two studies were not included [63,66]. Due to this, our findings disagree with the meta-analysis of both variables, and, regarding depression, the inclusion of these two studies, along with the recently published RCT by Yuenyongchaiwat, K (November, 2023) [69], probably suggest that niVR is not effective in reducing depressive symptoms in these patients.

The physical weakness, as well as the negative psychological impact (such as anxiety and depression) caused by CVD is associated with the poor level of QoL reported by these patients [74]. This meta-analysis assessed the effect of niVR on QoL, assessing not only the overall QoL but also the different dimensions of the QoL, such as physical, mental, social, and vitality. The effect of VR on overall QoL has only been assessed in the review of Blasco-Peris et al. (2022) [40] who were unable to find a statistical effect by which to improve it from data derived from three RCTs [63,66,68]. However, the overall finding was obtained by including repeated samples in the same analysis, which could limit their findings. In our analysis, although we included one more RCT [61], no statistically significant effect of niVR was shown on overall QoL and the physical, mental, social, and vitality dimensions.

The findings presented in this meta-analysis are clearly relevant for clinical practice for various reasons. Firstly, our findings support the use of niVR devices, such as Nintendo or Xbox plus Kinect, and active videogames as complementary physiotherapy tools in CRP to successfully improve aerobic capacity, cardiovascular endurance and anxiety. In comparison with other VR devices, such as immersive or semi-immersive VR devices, niVR presents some advantages. These devices are less expensive and enable the use of commercial video consoles for therapeutic objectives [75]. Additionally, this modality of VR presents a low risk of adverse events such as cyber sickness and temporo-spatial disorientation and allows for the training of all extremities in the therapy with controllers or sensors in upper and lower extremities. Of the nine RCTs included, only three [64,66,67] reported data on adverse events, estimating that approximately 4.8% of these patients could experience unwanted events performing niVR such as motion sickness, dyspnea, and fatigue. Furthermore, niVR active videogames are easier for patients to understand, encouraging adherence and motivation with the game [75,76,77]. In comparison with conventional therapies without niVR, patients have shown a higher level of satisfaction with the inclusion of niVR active videogames in CRP [63,64]. Lastly, all of these advantages suggest that niVR could be an excellent therapy tool for supervised tele-rehabilitation after hospital discharge, facilitating specific physical exercises during rehabilitation [78].

In highlighting the strengths of the present systematic review with meta-analysis, it is important to note some limitations. Firstly, the most important limitation is the low number of studies included in the variables assessed, especially in anxiety, which reduces the quality of the evidence and generalization of our findings. The second limitation is related to the large, medium, and low risk of performance, detection, and attrition biases, respectively, in the RCTs included, which could lead to underestimation or overestimation of the findings of these studies and of the meta-analysis. Another limitation is the negative impact produced by publication bias in the pooled effect of niVR for aerobic capacity. However, through the trim-and-fill estimation, we were able to estimate the adjusted pooled effect, indicating that the effect of niVR on aerobic capacity would be greater without publication bias. As a fourth limitation, we only assessed the effect of niVR on the variables of interest in the short-term (just at the end of the intervention), as the studies included did not provide enough data for medium or long-term follow-up. Finally, it is important to consider the heterogeneity between the protocols of application of niVR in the studies included, although statistical heterogeneity was not large in any meta-analysis. Future studies should aim to reduce the risk of bias, with the goal of including new studies in future meta-analyses so as to help clarify whether niVR would be effective in variables such as depression and QoL.

## 5. Conclusions

This meta-analysis is the first to exclusively assess the effect of niVR active videogames as part of CRP. It demonstrates that the use of niVR active videogames in cardiac rehabilitation is more effective than conventional cardiac rehabilitation and usual care in increasing aerobic capacity and cardiovascular endurance and reducing anxiety. However, in terms of improving depression and the various dimensions of QoL, such as physical, mental, social, vitality, and overall perceived health, no significant differences were observed between therapies. Moreover, this study confirms that niVR is a safe therapy for CRP, as evidenced by the low percentage of adverse events reported in the included studies. While this study supports the use of niVR in cardiac rehabilitation programs, it is essential that future studies be conducted with the aim of obtaining more robust conclusions with greater levels of evidence and precision.

## Figures and Tables

**Table 1:** Literature search strategies used in databases.

Database	Search Strategy
PubMed	(Cardiac Rehabilitation[mh] OR Cardiac Rehabilitation*[tiab] OR myocardial infarction[mh] or myocardial infarction[tiab] OR heart attack*[tiab]) AND (virtual reality[mh] OR virtual reality[tiab] OR virtual reality exposure therapy[mh] OR non-immersive virtual reality[tiab] OR “Nintendo”[tiab] OR “playstation”[tiab] OR “sony playstation”[tiab] OR “xbox”[tiab] OR Kinect[tiab] OR exergaming[mh] OR exergam*[tiab] OR video games[mh] OR video games[tiab] OR videogam*[tiab] OR “Wii”[tiab])
SCOPUS	(TITLE-ABS-KEY (“cardiac rehabilitation” OR “myocardial infraction” OR “heart attack”) AND TITLE-ABS-KEY (“virtual reality” OR “virtual reality exposure therapy” OR “non-immersive virtual reality” OR “video games” OR “videogames” OR “exergames” OR “exergaming” OR “Wii” OR “Nintendo” OR “playstation” OR “Kinect”))
WOS	(TOPIC(*cardiac rehabilitation* OR *myocardial infraction* OR *heart attack*) AND TOPIC (*virtual reality* OR *virtual reality exposure therapy* OR *non-immersive virtual reality* OR *video games* OR *videogames* OR *exergames* OR *exergaming* OR *Wii* OR *Nintendo* OR *playstation* OR *Kinect*))
CINAHL	(AB(cardiac rehabilitation OR myocardial infraction OR heart attack) AND AB (virtual reality OR virtual reality exposure therapy OR non-immersive virtual reality OR video games OR videogames OR exergames OR exergaming OR Wii OR Nintendo OR playstation OR Kinect))
SciELO	“Cardiac rehabilitation” AND (“virtual reality” OR exergame* OR videogame OR “video game”)
PEDro	(1) Cardiac rehabilitation AND virtual reality//(2) Heart attack AND Wii

**Table 2:** Characteristics of the studies included in the review.

Study	Participants (*n*; Pathology and Rehabilitation Phase)	NiVR Intervention				Control Intervention				Outcomes	
		*n*	Age	F:M	Intervention Characteristic	*n*	Age	F:M	Control Characteristic	Variable	Test
Brandao-Leandro, LA et al., 2021 (Brazil) [61] Setting: Local public health system Funding: yes	72 patients with controlled arterial hypertension in phase II	19	63.2 ± 10.3 years old	10:9	NiVR active videogames using Nintendo Wii (“Hula Hoop”, “Footing” and “Rhythmic Boxeo”) and Xbox 360 (“Run the Word-Broadway”, “Wall Breaker” and “Legs-100%”) during 70 min sessions, with 2 sessions per week over 15 weeks	20	66.5 ± 8.3 years old	12:8	Usual care	Functional capacity	6-MWT
						20	67.9 ± 6.4 years old	13:7	Aerobic exercise on treadmill over 12 weeks, three times per week and 50 min per session	Quality of life (overall, physical, mental, vitality, social)	SF-36
Cacau, LAP et al., 2013 (Brazil) [62] Setting: Fundaçao de Beneficiencia Hospital Cirujia, Aracaju, Sergipe. Funding: yes	60 patients with coronary artery bypass/valve replacement in phase I	30	49.2 ± 2.6 years old	17:13	Conventional physical therapy (breathing, metabolic and motor exercises and airway clearance techniques). NiVR was employed to perform motor exercises	30	52 ± 2.4 years old	14:16	Conventional physical therapy twice a day	Functional capacity	6-MWT
García-Bravo, S et al., 2020 (Spain) [63] Setting: cardiology service of the Hospital Universitario Fundación Alcorcón (Madrid) Funding: none	20 patients with ischemic heart disease in phase II	10	48.7 ± 9.9 years old	NR	40 min of aerobic and resistance exercises plus 20 min of niVR using Microsoft Xbox One and Kinect 2. Twice per week over 8 weeks	31	53.7 ± 10.3 years old	NR	Aerobic exercises plus resistance training for 60 min, twice per week over 8 weeks	Functional capacity	6-MWT
										Quality of life (overall, physical, mental, vitality, social)	SF-36
										Depression	BDI
Gulick, V et al., 2021 (United States) [64] Setting: Jefferson Health Methodist CR program, Philadelphia Funding: Yes	72 patients (mean age of 61 ± 9.9 years old and 20F:52M) with different cardiac diseases in phase II	41	-	-	Conventional physical therapy using Bionautica Trails VR System over 12 weeks	31	-	-	Conventional physical therapy over 12 weeks	Functional capacity	6-MWT
Jaarsma, T et al., 2021 (Sweden, Italy, Israel, Netherlands, Germany and United States) [65] Setting: NR Funding: yes	486 patients (33% female population) with heart failure in phase III	243	66 ± 12 years old	-	Nintendo Wii Sports (baseball, bowling, boxing, golf and tennis), five days per week, 30 min per session over one year	243	67 ± 11 years old	-	Usual care plus physical activity	Functional capacity	6-MWT
Ruivo, JMAS et al., 2017 (Ireland) [66] Setting: Kerry General Hospital Funding: none	32 participants with coronary artery bypass/valve replacement in phase II	16	59.4 ± 11.8 years old	2:14	60 min using Nintendo Wii Sports (boxing and canoeing), twice per week over 6 weeks	16	60.4 ± 8.5 years old	4:12	Conventional therapy, twice per week over 6 weeks	Quality of life (overall, physical, mental and social)	MNQ
										Anxiety	HADS
										Depression	
Silva, JPLN et al., 2018 (Brazil) [67] Setting: Physical Therapy Clinic of the Oeste Paulista University (Sao Paulo) Funding: yes	26 patients with different cardiac diseases in phase II	14	63.2 ± 8.3 years old	2:12	60 min using Microsoft Xbox 360 plus Kinect sensor (Your Shape and Dance Central 3), twice per week over 8 weeks	12	63.8 ± 8.7 years old	6:6	Aerobic exercise using treadmill for 30 min and strength training	Functional capacity	
Vieira, A et al., 2018 (Portugal) [68] Setting: Hospital Centre of Porto Funding: none	33 patients with different cardiac diseases in phase III	11	55 ± 9 years old	-	NiVR using Kinect (Kinect-Rehab play project) plus education of control of risk factors in sessions of 60 min, three times per week for 24 weeks	11	59 ± 11.3 years old		Education plus exercise training	Quality of life (overall, physical, mental and social)	MNQ
						11	59 ± 5.8 years old		Education plus usual care and daily walks		
										Anxiety	DASS-21
										Depression	
Yuenyongchaiwat, K et al., 2023 (Thailand) [69] Setting: University Hospital (Khlong Nueng) Funding: yes	60 patients with coronary heart or valvular disease	30	63.2 ± 9.6 years old	14:16	NiVR using Toucher Software (“Falling Snow”, “Apple Tree” and “Hit the mole”) for 30 min, once a day until hospital discharge	30	64.4 ± 8.7 years old	11:19	30 min of conventional physical therapy, once a day until hospital discharge	Functional capacity	6-MWT
										Depression	PHQ-9

Abbreviations: niVR, non-immersive virtual reality; n, number of participants; F, female; M, male; NR, non-reported data; min, minutes; 6-MWT, 6-Minute Walking Test; BDI, Beck Depression Inventory; HADS, Hospital Anxiety and Depression Scale; MNQ, MacNew Questionnaire; DASS-21, Depression, Anxiety and Stress Scale 21; PHQ-9, Patient Health Questionnaire 9.

## Data Availability

The dataset analyzed in the current study is available from the corresponding author on reasonable request.

## Supplementary Materials

The following supporting information can be downloaded at: https://www.mdpi.com/article/10.3390/s24030903/s1, Figure S1: Funnel plot for aerobic capacity and cardiovascular endurance; Table S1. Mean bias risk score of the individual studies.
