# Supplementary material for: Non-Immersive Virtual Reality-Based Therapy Applied in Cardiac Rehabilitation: A Systematic Review with Meta-Analysis
Source: Sensors (Basel). 2024 Jan 30;24(3):903. doi: 10.3390/s24030903 (PMC10857255; doi:10.3390/s24030903)
Supplement: Supplementary file 1 [file sensors-24-00903-s001.zip › sensors-2793498-supplementary.pdf]

SUPPLEMENTARY MATERIAL

Supplementary Table S1. Mean bias risk score of the individual studies.

Study	Bias Risk Score
Brandao-Leandro, LA et al 2021	78%
Cacau, L et al 2013	71%
García-Bravo, S et al 2020	78%
Gulick, V et al 2021	57%
Jaarsma, T et al 2021	78%
Ruivo, JMAS et al 2017	78%
Silva, JPLN et al 2018	71%
Vieira, A et al 2018	71%
Yuenyongchaiwat, K et al 2023	71%
**Mean Score**	**72% (±6.8%)**

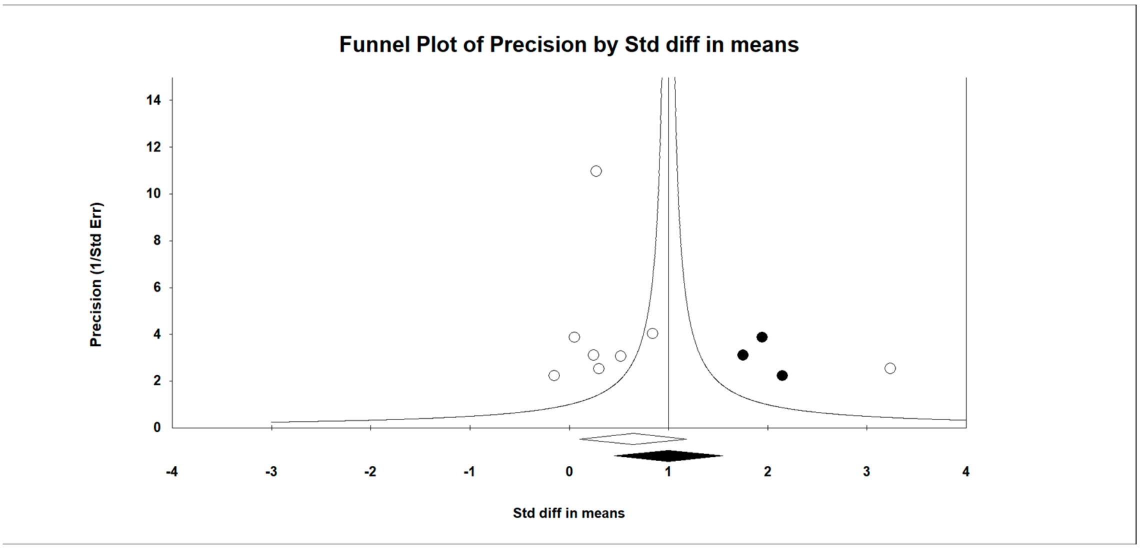

Figure S1. Funnel plot for aerobic capacity and cardiovascular endurance (physical function)
